# Patient's decision and experience in the multi-channel appointment context: An empirical study

**DOI:** 10.3389/fpubh.2022.923661

**Published:** 2022-08-01

**Authors:** Qing Ye, Hong Wu

**Affiliations:** ^1^Tongji Hospital, Tongji Medical College, Huazhong University of Science and Technology, Wuhan, China; ^2^School of Medicine and Health Management, Tongji Medical College, Huazhong University of Science and Technology, Wuhan, China

**Keywords:** patient experience, waiting time, multi-channel context, health-related factor, cost-related factor

## Abstract

**Background:**

Long waiting time for treatment in the outpatient department has long been a complaint and has influenced patient's experience. It is critical to schedule patients for doctors to reduce patient's waiting time. Nowadays, multi-channel appointment has been provided for patients to get medical services, especially for those with severe illnesses and remote distance. This study aims to explore the factors that influence patient appointment channel choice in the context of multi-channel appointments, and how channel choice affects the waiting time for offline visiting.

**Methods:**

We collected outpatient appointment records from both online and offline appointment channels to conduct our empirical research. The empirical analysis is conducted in two steps. We first analyze the relationship between appointment channel choice and patient's waiting time and then the relationships between three determinants and appointment channel choice. The ordinary least squares and the logistic regression model are used to obtain the empirical results.

**Results:**

Our results show that a patient with an online appointment decision has a shorter consultation waiting time compared with a patient with on-site appointment (β = −0.320, *p* < 0.001). High-quality resource demand (β = 0.349, *p* < 0.001), high-severity disease (β = 0.011, *p* < 0.001), and high non-disease costs (β = 0.039, *p* < 0.001) create an obvious incentive for patients to make appointments *via* the Internet. Further, only the effect of non-disease cost on channel choice is lower for patients with multiple visit histories (β = −0.021, *p* < 0.001).

**Conclusions:**

Our study confirms the effect of Internet use on reducing patient's waiting time. Patients consider both health-related risk factors and cost-related risk factors to make decisions on appointment channels. Our study produces several insights, which have implications for channel choice and patient's behavior literature. More importantly, these insights contribute to the design of appointment systems in hospitals.

## Introduction

Patient's waiting time refers to the length of time from when the patient entered the waiting room or the consulting room to the time the patient received the services and left the doctor's consulting room, is closely related to the willingness to return for care and satisfaction ratings (1, 2), and affects the utilization of healthcare services (3). Patients may be less able to judge the technical quality of the care they receive, but they do judge their social interaction with the doctors (4). Among them, waiting time is usually regarded as indicator of service quality (5). Some patients even wait in line all night to ensure registration with a certain doctor (6). A long unnecessary waiting time can be a cause of stress for both patient and doctor (2, 7). Failure to incorporate patient-driven features into the design of service could lead to disharmonious patient–provider relationships (2).

Prior studies indicated that a key anecdotal source of dissatisfaction with medical services reported by patients is having to wait a long time in the office (1, 2). So, time spent waiting before the consultation has attracted much research attention, and researchers begin to explore the determinants of patient's waiting time (3, 8), including few healthcare workers, a large number of patients, and the use of computers. Among these factors, an effective appointment system is a critical component in controlling patient's waiting time (9). Researchers have simulated various appointment schedules by considering patient types and varied care needs and analyzed the corresponding patient's waiting times (9–12). However, these designs are difficult to generalize in practice because of the difficulty of implementing optimization models in healthcare systems, especially in China. In addition, patient's behavior and decision-making create greater uncertainty about waiting times (13, 14).

With the development of e-health, various medical services have been provided *via* the Internet and attracted a great number of patients (15–17). Among these services, the online appointment service is generally embraced by patients. Using survey data, the prior study has found that the online appointment system can significantly reduce patient's waiting time compared with the usual queueing method (6). To date, there are few studies about the efficacy of online appointments on reducing patient's waiting time that are conducted using a big sample size and real operation data. Most existing studies depend on the survey data [e.g., (6)]. In addition, although the benefits of the online appointment channel using have been recognized, its determinants have not been fully understood. In our previous study, we explored the impact of external resource status on patient mHealth adoption but did not delve into the influences of patient channel choice and the impact on waiting time (17). By identifying the determinants of patients' channel choice, hospitals can develop intervention strategies to further improve the usability of online channels.

Due to the limited and uneven distribution of medical resources in China, long waiting time for consultation is common in the healthcare system and seriously influence the patient's experience. Whereas previous studies have examined the issues such as factors that influence patient mHealth adoption and patient's experience, there are no studies that have considered patient service channel choice and the impact on patient's offline waiting time. Patient-centered health care aims to improve medical resource accessibility and user experience through information technology, as a part of the Healthy China strategy, which the Chinese government has already taken action on. To fill these gaps in existing research and practice, this study investigates the antecedents and consequences of patients' appointment decisions in the general outpatient department under the multi-channel appointment context by collecting a real dataset from a tertiary hospital in China. The specific research questions being addressed in this paper are as follows:

1) What is the average patient's waiting time in the consulting room?
2) Whether the appointment channel will affect patient's waiting time? And how?
3) What factors will influence the patient's decision on the choice of appointment channel?

The real operation data from 1,241 doctors from 119 departments, involving 308,085 patients, were used to answer these questions. This study is among the first to examine the relationship between appointment channel and patient's experience that is measured by patient's waiting time and the determinants of appointment channel selection. The empirical results provide a basis for theorizing the channel choice in the new context and these insights contribute to the designs of the appointment systems in hospitals.

## Theoretical background and hypotheses development

### Experience of waiting time in the outpatient department

**Figure 1:**
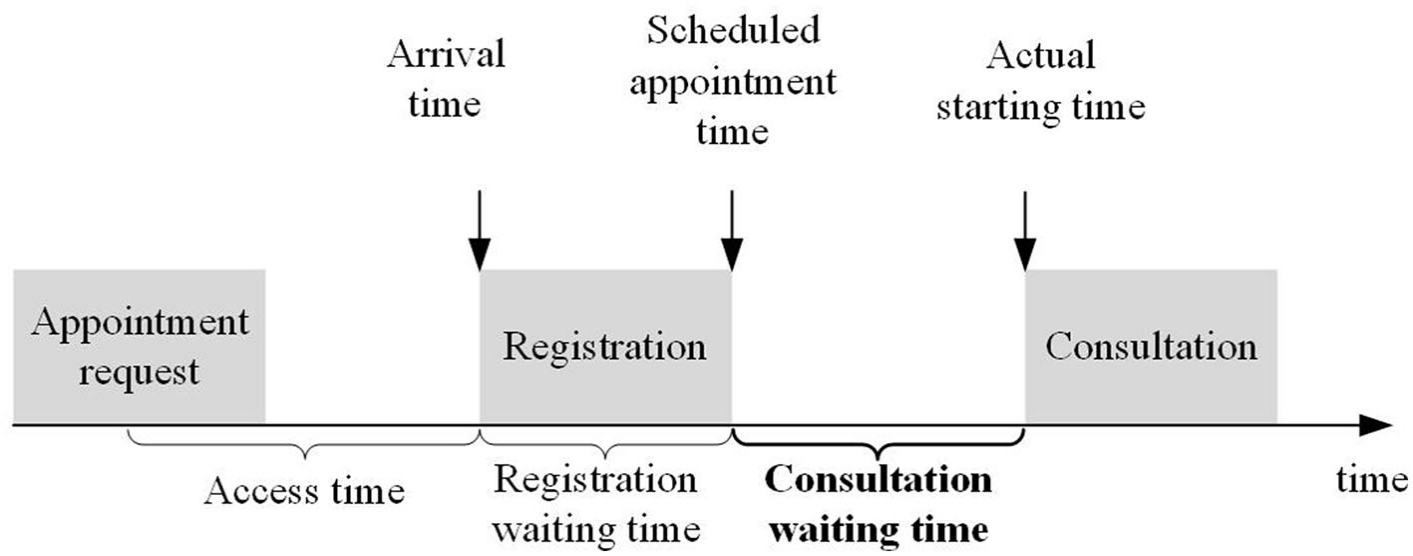
The general patient flow for getting medical services.

Waiting time in the outpatient department is directly related to the patient's satisfaction with the medical services received. Long waiting time is a generally existent phenomenon in China as medical resources are limited. Figure 1 describes the general patient flow from appointment request to the moment of consultation (18). Once patients decide to obtain medical services in the hospitals, they must go through the registration process and consultation process. The waiting time can be divided into the following distinct categories:

1) The registration waiting time. It measures the length of waiting time for registration. In the registration process, a patient who has chosen the offline appointment channel—on-site appointment—is required to queue up for filling in registration forms or presenting an identification card to the registration staff and designate a department or a doctor and then get a queue number for consultation. For a patient with an online appointment, s/he has to make an appointment based on the doctor's available dates *via* the Internet and show his/her appointment information and identification card to get a queue number for consultation in the hospital. Therefore, the processes of registration for making appointments online and offline are different.
2) The consultation waiting time. It measures the length of waiting time for consultation, namely, the waiting time between scheduled appointment time and the actual starting time. As patients with online and offline appointments have the same operation processes, the waiting time for the consultation is included in this study.

The registration waiting time for outpatients has already become a long-festering healthcare problem in China and has been fully studied (6). Compared with the registration waiting time, the consultation waiting time is often overlooked. In addition, the processes of registration for making appointments online and offline are different; therefore, our study focuses on consultation waiting time only, namely, the waiting time between scheduled appointment time and actual starting time.

### Appointment channel and patient's waiting time

Outpatient services are an important component of health care and influence patient's satisfaction (19, 20). Long waiting time for treatment in the outpatient department has long been a complaint (21) and is a critical determinant of patients' choices in hospitals (22). In many service industries, the waiting time influences consumers' service experience and they often use the waiting time as a decisive factor in choosing a service provider (23). Therefore, researchers emphasized that the waiting time must be considered in designing an appointment system.

Existing studies mainly focus on how to design an appointment system to reduce the patient's waiting time (24) or no-show behavior (25). A longer waiting time relates to reneging behavior (23). Appointment scheduling systems are widely used by medical service providers to regulate their service capacity and demands. Providing pre-scheduled appointments helps to reduce the variability in demands and allows providers to better play their operations (25). The outpatient appointment service is provided through both online and offline channels. Online channels include the WeChat platform, APP, and third-party platforms such as haodf.com and Chutian mingyi platform, and offline channels include manual window service. To compare the differences between online and offline channels, we merge the multiple channels according to the online and offline dimensions. Using the online appointment channel, the medical services can be pre-scheduled, which brings benefits for both patients and doctors. Using a pre-scheduling mechanism, doctors can regulate their service capacity and balance the demands between online and offline channels, which helps to avoid overload situations. The overload of doctors is the main factor that influences patient's waiting time. Hence, we have:

**H1**. Compared with offline appointment, patients with online appointments have a lower length of waiting time for consultation.

### Determinants of appointment channel choice

Although the Internet has radically changed public service delivery, the use of traditional service channels remains high, especially in health care. Based on the Media Richness Theory (26), media differ in richness and have different capacities to provide cues. Compared with the offline appointment channel, patients can get more information *via* the online appointment channel and chances to choose a satisfied doctor.

The area of human–computer interaction has also discussed channel choice. The perceived accessibility and quality of information sources significantly influence channel choice (27). In addition, perceived usefulness and perceived ease of use are the primary relevance for computer acceptance behaviors based on the Technology Acceptance Model (28, 29). In marketing, the impact factors of channel choice have also been widely explored, with perceived risk, propensity, convenience, transaction costs, ease of use, complexity, trust, and flexibility which are the main factors discussed in existing studies (30). However, in no other field than marketing, channel choice received so much attention.

From the analysis of existing research in other fields, we can draw a major conclusion: we lack an understanding of what factors are relevant in the healthcare context. In health care, health-related risk factors and cost-related risk factors are two critical major concerns of patients (31, 32). For the health-related factors, since medical services deal with life and wellness, patients are eager to find high-quality physicians (4, 17, 33). For the cost-related factors, cost plays a vital role for consumers in deciding from whom to get the products/services, and higher costs decrease demand and increase switching in most circumstances (34). Patients in medical institutions show a very significant geographic distribution trend, which is also practical proof that cost-related factors affect patient service choices (17).

For the relationships between health-/cost-related factors and the patient's appointment choice, we conduct the following analysis. First, when the situation gets more ambiguous, people would try to find more reliable information sources to reduce uncertainty (35, 36). Therefore, when patients get serious diseases and need to find a high-quality (scarce) medical resource, they would tend to make appointments *via* the Internet as the Internet can provide certain results. Second, effort is the most important determinant of channel choice, namely people tend to choose the most convenient channel (17). The online channel provides more information conveniently and helps patients to make a satisfying choice easily, which could reduce the possibility of failing to choose a satisfied doctor and high costs. In this paper, resource type demand and disease severity are used to measure the health-related risk factors, and non-disease costs (including time cost, transportation cost, housing cost, etc.) are used to represent the cost-related risk factors (37, 38). Hence, we have:

**Health-related risk factors:**

**H2a:** High-quality resource demand is positively related to the online appointment choice.
**H2b:** High-severity disease is positively related to the online appointment choice.

**Cost-related risk factor:**

**H2c:** High non-disease cost is positively related to the online appointment choice.

### The moderating effects of patients' visiting history

Perceived self-efficacy in the Theory of Planned Behavior has demonstrated people's judgment of their capabilities to organize and execute actions to attain designated performance (39). Both familiarity and domain expertise contribute to consumer's knowledge and influence their decision-making ability (40, 41). Internet experience creates a great sense of comfort with the online channel and thereby helps to reduce perceived uncertainty and increase decision-making ability (42).

Consumer behaviors change over time since the experience increases from past purchases (43). When consumers repeat purchase behavior several times, they feel more and more in control and change behavior correspondingly (44). Prior study has identified the moderating effect of prior experience on consumer's behavior (42). In this study, we use visiting history to represent patients' appointment channel choice experience. Each offline visit will be experienced as a channel choice. Therefore, we propose that when patients have a visit history in the hospital, they are familiar with the operation process and have low uncertainty, leading us to the following hypotheses:

**H3a:** Visiting history decreases the positive impact of resource type demand on online appointment choice.
**H3b:** Visiting history decreases the positive impact of the severity of diseases on online appointment choice.
**H3c:** Visiting history decreases the positive impact of the non-disease costs on online appointment choice.

**Figure 2:**
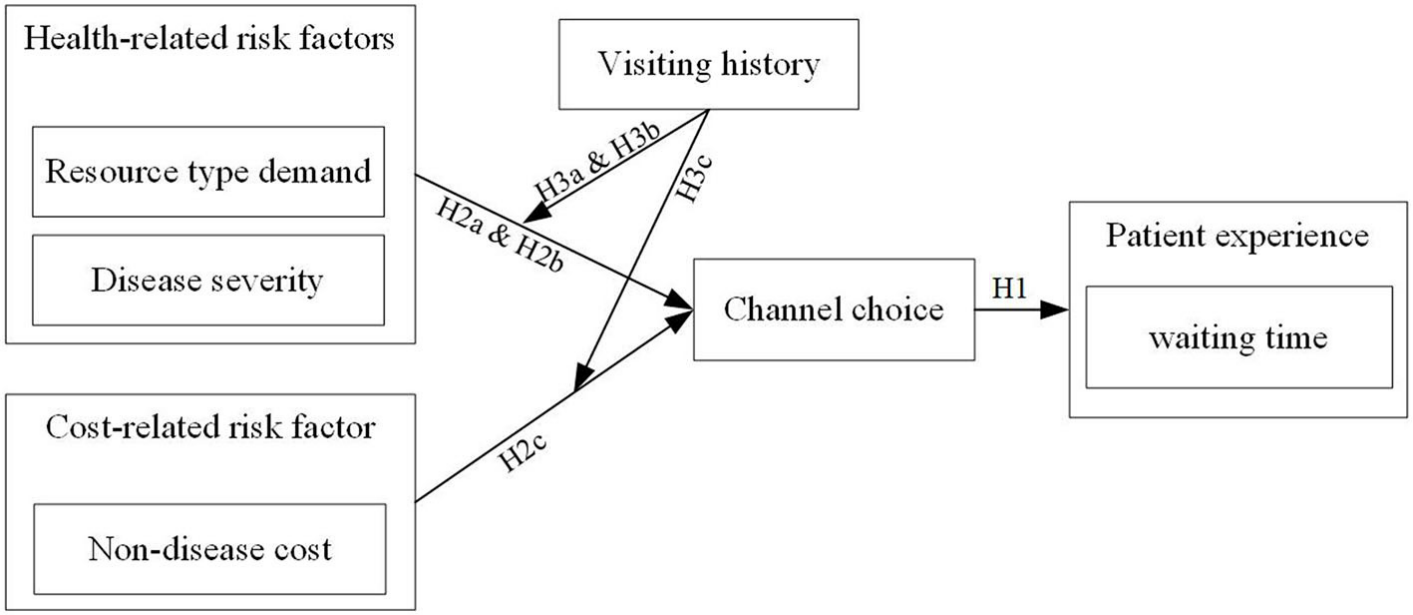
The conceptual model.

Figure 2 shows our conceptual research model.

## Methods

In this section, we describe our research context, data collection process, variables, and empirical models.

### The research context and data collection

To answer the research questions, we collect a real dataset from a tertiary hospital in China which has been founded over 100 years ago. This hospital began to implement the online appointment service since 2014. Patients can make appointments *via* WeChat, the hospital's APP, and some third-party platforms. Besides the online appointment channel, patients can also make appointments *via* the traditional offline channel. As the hospital has superior doctors, advanced medical equipment, and technology, it has attracted many patients from all over the country, which provides a wide range of distance between the patient location and the hospital location and helps us to explore the impact of distance. The hospital has recorded sources for each patient, which helps us to distinguish appointment channels for each patient, and measure health-related risk factors, cost-related risk factors, and visiting history. In 2019, about one-third of the patients have made appointments *via* the Internet.

We extracted data from outpatient appointment records and collected all outpatient data for January 2019 to conduct our empirical research. Specifically, we used structured query language (SQL) query statements to retrieve data from the database, exported them to CSV format, and finally imported them to Rstudio version 1.2 for data cleaning and processing. Since there are many online channels and they are changing all the time, there is no uniform online appointment format template, and all of them interact with the backend database through the web interface, but the collected information includes all the data needed for this research. According to our problem, our data screening and processing procedures followed the following rules: First, patients in the emergency department were removed because emergency patients' waiting time was not subjected to various rules and were not eligible for this study. Second, we removed all no-show patients, who were not in the queue. Third, we removed anomalous data with a waiting time of more than 24 h, which accounts for a relatively small proportion of 1.9% and thus has less impact on our findings. Fourth, since the patient's channel selection and waiting time for each visit are independent, multiple visits of patients will be included in the study as multiple samples. Finally, the real operation data from 1,241 doctors from 119 departments, involving 308,085 patients from both online and offline appointment channels, were collected. The dataset contains information on the use of appointment channels for outpatient visits, demographic characteristics of patients, and disease-related information.

### Variables and models

The definitions for all variables used in this study are presented in Table 1.

**Table 1:** Variable definitions.

**Variables**	**Symbols**	**Coding**
Waiting time	*WT*	The logarithm of the consultation waiting time (in minutes) of patients.
Appointment channel	*CHANNEL*	Appointment channels for patients, offline is coded as 0 and online as 1.
Resource type demand	*RES_TYPE*	The title of the doctor that patient visit, 1 is the chief doctor, 0 is the associate chief doctor.
Disease severity	*SEVERITY*	The logarithm of the total cost of the current patient visit.
Non-disease cost	*NonD_COST*	The distance from patients to the clinic. Zero if the patient is in the city where the hospital is located and otherwise 1.
Visiting history	*HISTORY*	The logarithm of the number of previous visits.
Gender	*GENDER*	Males are coded as 0 and females as 1.
Age	*AGE*	Three dummy variables are used to measure the age of patients, AGE1 represents patients between 18 and 45 years old, AGE2 represents patients between 46 and 59 years old and AGE3 represents patients above 60 years old.
SITE	*SITE*	The hospital has three sites in the city, and two dummy variables are used to measure it.
Hour of day	*HOUR_ DAY*	The periods of patients want to see a doctor, including all-day, morning, and afternoon. Two dummy variables are used to measure it. HOUR_DAY1 represents morning and HOUR_DAY2 represents afternoon.

#### Patient's waiting time (WT)

It measures the length of consultation waiting time, namely, the waiting time between scheduled appointment time and actual starting time.

#### Appointment channel choice (CHANNEL)

A dummy variable is set to measure a patient's appointment channel choice. Zero represents the traditional on-site appointment and 1 represents appointment *via* the Internet, including WeChat, the hospital's APP, and third-party platforms.

#### Health-related risk factors

The resource type demand and severity of diseases are included to indicate health-related risk factors. The doctor's title is used to measure resource type demand (*RES_TYPE*), with the chief doctor representing high-quality resources. The total cost of a patient in the hospital is used to measure the severity of disease (*SEVERITY*).

#### Cost-related risk factor

The non-disease cost (*NonD_COST*) is measured, including time cost, transportation cost, housing cost, etc. The distance between the patient location and the hospital location is calculated to measure the non-disease cost. Specifically, a dummy variable is set for 0 if the patient is in the city where the hospital is located, and otherwise 1.

#### Visiting history (HISTORY)

It measures the number of patients' previous visits to the hospital.

#### Control variables

Prior studies have proved that demographic characteristics have significant impacts on consumers' preferences for different channels (45, 46). Therefore, gender, age, and site are included to control the model. In addition, the hour of the day that measures the period for the appointment is also included. Detailed descriptions can be found in Table 1.

The empirical analysis is conducted in two steps. We first analyze the relationship between appointment channel choice and patient's waiting time and then the relationships between three determinants and appointment channel choice. We used the lm function from the stats package in R language to fit our model.

**Step 1**. To test H1, a linear model was employed to estimate the effect of appointment channel choice on patient's waiting time. The ordinary least squares method was used to fit our models. The models were specified as follows:


$$\begin{array}{l} WT_{i}=\;\beta_{0}+\beta_{1}CHANNEL_{i}+\beta_{2}AGE_{i}+\beta_{3}GENDER_{i}\\ \;+\beta_{4}SITE_{i}+\beta_{5}HOUR\_DAY_{i}+\varepsilon_{i} \end{array}$$

**Step 2**. Since CHANNEL is a binary variable, a logistic regression model was used to estimate the effect of three determinants on appointment channel choice. The multiple regression model is as follows:


$$\begin{array}{l} CHANNEL_{i}=\;\beta^{\prime }{}_{0}+\beta^{\prime }{}_{1}RES\_TYPE_{i}+\beta^{\prime }{}_{2}SEVERITY_{i}\\ \;+\beta^{\prime }{}_{3}NonD\_COST_{i}+\beta^{\prime }{}_{4}HISTORY_{i}\\ \;+\beta^{\prime }{}_{5}RES\_TYPE_{i}\times HISTORY_{i}+\beta^{\prime }{}_{6}SEVERITY_{i}\\ \;\times HISTORY_{i}+\beta^{\prime }{}_{7}NonD\_COST_{i}\times HISTORY_{i}\\ \;+\beta^{\prime }{}_{8}AGE_{i}+\beta^{\prime }{}_{9}GENDER_{i}+\beta^{{}^{\prime }}{}_{10}SITE_{i}\\ \;+\beta^{\prime }{}_{11}HOUR\_DAY_{i}+\varepsilon^{\prime }{}_{i} \end{array}$$

where *i*=1,…,n indexes the patient. β are coefficients needed to be estimated. *RES_TYPE*×*HISTORY, SEVERITY*×*HISTORY*, and *NonD_COST*×*HISTORY* are interaction items. ε is the error term.

## Results

The ordinary least squares and the logistic regression model are used to obtain empirical results. All our empirical models are done in RStudio version 1.2.

### Descriptive statistic

Descriptive statistics for the key variables used in the analysis are presented in Tables 2–5. The average waiting time is 99.47 min, with 92.18 min for patients with the online appointment channel and 114.55 min for ones with the offline appointment channel. Nearly two-thirds of patients make appointments *via* the traditional on-site channel. The distribution of the doctors across various titles such as the chief doctor and associate chief doctor was even at approximately 60 and 40%. More than one-third of patients come from outside the city where the hospital locates. The average visiting time is 7.10 min. We find that there are 62.3% [=51.2/(51.2+31.4)] of patients want to see a doctor in the morning. The appointment channel is related to patient's waiting time, and three determinants are also related to patients' appointment channel choice. Also, the correlations between the independent variables and control variables are low, which helps yield stable results.

**Table 2:** The descriptive statistics of waiting time for different groups.

	**Average waiting time (min)**	**Observations**
Total samples	99.47	308,085
Samples with online appointment channel	92.18	100,383
Samples with offline appointment channel	114.55	207,702

**Table 3:** Descriptive statistics for variables (*n* = 308,085).

**Measure**	**Mean**	**St. Dev**.
*Ln(WT)/WT*	3.965/99.47 min	1.273
*CHANNEL* (offline, online)	(0.674, 0.326)	
*RES_TYPE* (associate chief doctor, chief doctor)	(0.400, 0.600)	
*Ln(SEVERITY)*	5.302	1.639
*NonD_COST* (in the city, others)	(0.605, 0.395)	
*Ln(HISTORY)/HISTORY*	1.677/7.10	0.848
*GENDER* (male, female)	(0.426, 0.574)	
*AGE* (between 18 and 45 years old, between 46 and 59 years old and above 60 years old.)	(0.476, 0.233, 0.133)	
*SITE* (site 1, 2 and 3)	(0.732, 0.158, 0.111)	
*HOUR_DAY* (all day, morning, and afternoon)	(0.168, 0.518, 0.314)	

**Table 4:** Correlations of variables.

**Variables**	**1**	**2**	**3**	**4**	**5**	**6**	**7**	**8**	**9**
1. *WT*									
2. *CHANNEL*	−0.206***								
3. *RES_TYPE*	0.348***	0.375***							
4. *SEVERITY*	−0.037***	0.038***	0.053***						
5. *NonD_COST*	0.071***	0.037***	0.092***	0.034***					
6. *HISTORY*	−0.039***	−0.089***	−0.079***	0.067***	−0.083***				
7. *GENDER*	−0.005***	0.006***	−0.021***	−0.006***	−0.024***	0.076***			
8. *AGE*	−0.059***	−0.074***	0.004**	0.036***	0.044***	0.007***	0.024***		
9. *SITE*	−0.041***	−0.052***	−0.108***	−0.053***	−0.171***	0.047***	−0.012***	0.012***	
10. *HOUR_DAY*	0.366***	0.173***	0.384***	−0.060***	−0.015***	−0.085***	−0.067***	−0.086***	0.187***

^*^p < 0.05;

^**^p < 0.01;

^***^p < 0.001.

**Table 5:** Results for appointment channel-patient's waiting time.

	**WT**	
	**Model 1**	**Model 2**
CHANNEL		−0.320*** (0.005)
GENDER	0.088*** (0.004)	0.079*** (0.004)
AGE1	−0.287*** (0.006)	−0.272*** (0.006)
AGE2	−0.175*** (0.007)	−0.151*** (0.007)
AGE3	−0.209*** (0.008)	−0.184*** (0.008)
SITE1	−0.404*** (0.006)	−0.367*** (0.006)
SITE2	−0.401*** (0.007)	−0.351*** (0.007)
HOUR_DAY1	1.020*** (0.006)	0.902*** (0.008)
HOUR_DAY2	1.532*** (0.007)	1.425*** (0.008)
Adjusted R2	0.166	0.179
Residual Std. Error	1.163 (df = 308,076)	1.154 (df = 308,072)
F Statistic	7,665.500*** (df = 8; 308,076)	7,450.886*** (df = 9; 308,072)

^*^p < 0.05;

^**^p < 0.01;

^***^p < 0.001.

### Data analysis

Empirical results are shown in Tables 5, 6. To make sure that the results are not driven by multicollinearity, we gradually added in different sets of independent and control variables.

**Table 6:** Results for three determinants-appointment channel.

	**CHANNEL**		
	**Model 1**	**Model 2**	**Model 3**
RES_TYPE		0.312***	0.349***
		(0.002)	(0.004)
SEVERITY		0.006***	0.011***
		(0.0005)	(0.001)
NonD_COST		0.008***	0.039***
		(0.002)	(0.004)
HISTORY		−0.032***	−0.002
		(0.001)	(0.003)
GENDER	0.028***	0.024***	0.028***
	(0.002)	(0.002)	(0.002)
AGE1	−0.046***	−0.069***	−0.069***
	(0.002)	(0.002)	(0.002)
AGE2	−0.075***	−0.109***	−0.110***
	(0.003)	(0.003)	(0.003)
AGE3	−0.077***	−0.108***	−0.109***
	(0.003)	(0.003)	(0.003)
SITE1	−0.115***	−0.017***	−0.014***
	(0.002)	(0.002)	(0.002)
SITE2	−0.156***	−0.051***	−0.045***
	(0.003)	(0.003)	(0.003)
HOUR_DAY1	0.369***	0.104***	0.097***
	(0.002)	(0.003)	(0.003)
HOUR_DAY2	0.335***	0.086***	0.080***
	(0.003)	(0.003)	(0.003)
RES_TYPE × HISTORY			−0.025
			(0.003)
SEVERITY × HISTORY			−0.002
			(0.001)
NonD_COST × HISTORY			−0.021***
			(0.002)
Adjusted R^2^	0.084	0.152	0.156
Residual Std. Error	0.448 (df = 308,076)	0.432 (df = 308,073)	0.431 (df = 308,069)
F Statistic	3,554.217*** (df = 8; 308,076)	5,027.065*** (df = 11; 308,073)	3,801.954*** (df = 15; 308,069)

^*^p < 0.05;

^**^p < 0.01;

^***^p < 0.001.

#### Step 1. Results for the relationship between appointment channel choice and patient's waiting time

From Table 5, we find that making appointments *via* the Internet can significantly decrease patient's waiting time (β = −0.320, *p* < 0.001), the average reduced waiting time is 1.38 min. Therefore, H1 is supported.

#### Step 2. Results for the relationship between three determinants and appointment channel choice

Table 6 shows the impacts of health-related risk factors and cost-related risk factors on patients' appointment channel choices. We find that high-quality resource demand (β = 0.349, *p* < 0.001), the severity of disease (β = 0.011, *p* < 0.001), and the non-disease costs (β = 0.039, *p* < 0.001) positively improve patients' propensity to make appointments *via* the Internet. Among these three determinants, the influence of high-quality resource demand is the biggest. Therefore, H2a–c are supported.

For the moderating effects of patients' visiting history, we find that compared with patients without visiting history in the hospital, the impact of non-disease costs on appointment channel choice is small for patients with visiting history (β = −0.021, *p* < 0.001). However, no significant results are found for the moderating effects of visiting history on relationships between health-related risk factors (resource-type demand and severity of disease) and appointment channel choice. Therefore, H3a–b are not supported, and H3c is supported.

### Heterogeneity tests

Based on the main results in Table 6, we find the impacts of independent variables and control variables are quite significant. Therefore, we further examine the heterogeneity of different patient groups. We divided patient samples based on gender, the period, and resource type demand and obtain the empirical results (shown in Table 7). We find that most results for independent variables are consistent with our main results. An interesting result is found for the male group. For male patients, they tend to make appointments directly on-site when getting serious diseases. The possible explanation is that males and females adopt different strategies in decision environments males are more risk-seeking than females (47).

**Table 7:** Heterogeneity test results for appointment choice.

	**Gender**		**HOUR_DAY**		**Resource type**	
	**Male**	**Female**	**Morning**	**Afternoon**	**Chief doctor**	**Associate chief doctor**
RES_TYPE	0.429***	0.355***	0.374***	0.352***		
	(0.015)	(0.005)	(0.006)	(0.007)		
SEVERITY	−0.065***	0.013***	0.008***	0.013***	0.018***	0.010***
	(0.004)	(0.001)	(0.002)	(0.002)	(0.001)	(0.001)
NonD_COST	0.178***	0.041***	0.044***	0.039***	0.062***	0.005
	(0.015)	(0.005)	(0.005)	(0.007)	(0.005)	(0.004)
RES_TYPE × HISTORY	−0.031	−0.023	−0.043	−0.012		
	(0.015)	(0.004)	(0.005)	(0.006)		
SEVERITY × HISTORY	0.015	−0.003	−0.0001	−0.0005	−0.005	−0.003
	(0.002)	(0.001)	(0.001)	(0.001)	(0.0003)	(0.0002)
NonD_COST × HISTORY	−0.031***	−0.024***	−0.024***	−0.023***	−0.029***	−0.011***
	(0.008)	(0.002)	(0.003)	(0.004)	(0.003)	(0.002)
Observations	131,406	176,679	159,558	96,753	184,821	123,264
Adjusted R^2^	0.206	0.167	0.099	0.110	0.011	0.098
Residual Std. Error	1.149 (df = 131,391)	0.429 (df = 176,664)	0.463 (df = 159,544)	0.453 (df = 96,739)	0.496 (df = 184,808)	0.298 (df = 123,251)
F Statistic	2,429.348*** (df = 14; 131,391)	2,523.347*** (df = 14; 176,664)	1,346.869*** (df = 13; 159,544)	921.329*** (df = 13; 96,739)	172.335*** (df = 12; 184,808)	1,122.707*** (df = 12; 123,251)

^*^p < 0.05;

^**^p < 0.01;

^***^p < 0.001.

Results for control variables are omitted.

### Robustness checks

In the main analysis, one month of data was collected. To check the robustness of our results, we collected a new dataset with a 3-month interval ranging from January 2019 to March 2019 and used the new data to obtain empirical results (shown in Table 8). Consistent results are found, and the results appear to be robust.

**Table 8:** Robustness check results.

	**WT**		**CHANNEL**	
	**Model 1**	**Model 2**	**Model 2**	**Model 3**
CHANNEL		0.185***		
		(0.003)		
RES_TYPE	0.622***	0.558***	0.344***	0.384***
	(0.003)	(0.003)	(0.001)	(0.002)
SEVERITY	−0.044***	−0.044***	0.004***	0.009***
	(0.001)	(0.001)	(0.0003)	(0.001)
NonD_COST	0.139***	0.138***	0.007***	0.039***
	(0.003)	(0.003)	(0.001)	(0.002)
RES_TYPE × HISTORY				−0.027
				(0.002)
SEVERITY × HISTORY				−0.002
				(0.0003)
NonD_COST × HISTORY				−0.023***
				(0.001)
Adjusted R^2^	0.165	0.169	0.166	0.171
Residual Std. Error	1.144 (df = 850,124)	1.141 (df = 850,123)	0.429 (df = 850,124)	0.428 (df = 850,120)
F Statistic	1.144 (df = 850,124)	14,447.190*** (df = 12; 850,123)	15,430.130*** (df = 11; 850,124)	11,710.860*** (df = 15; 850,120)

^*^p < 0.05;

^**^p < 0.01;

^***^p < 0.001.

Results for control variables are omitted.

## Discussion and implications

To the best of our knowledge, our study is among the first that tests the effects of appointment channels (both online and offline) on patient's experience measured by patient's waiting time and investigates the determinants of channel choice in health care. Although the literature on online appointment is abundant, they all explore the factors that influence the adoption of mHealth and the impact of mHealth from a local perspective, without using a global perspective to delve into the influencing factors and mechanisms of action of the online channel in improving the patient's experience. Our study integrates previous research findings, conducts empirical studies based on a large amount of observational data, and provides new insights. Our findings have theoretical and practical support for policymakers and healthcare providers to promote mHealth services, improve service delivery, and enhance the patient's experience. In addition, our findings can help relevant people understand patient's appointment behavior.

### Result analysis

Using a real dataset from a tertiary hospital in China, we find strong and robust evidence of the antecedents and consequences of channel choice. Our results confirm the effect of Internet use on reducing patient's waiting time. Patients consider both health-related risk factors and cost-related risk factors to make decisions on appointment channels, which is consistent with prior studies (30). Our empirical study generates several important results.

Patients who make appointments *via* the Internet have a shorter waiting time. Waiting time is a decisive factor in choosing service providers (23). Long waiting time is a common phenomenon in hospitals, especially these tertiary hospitals, and needs to be considered in designing appointment systems. Our results show that using the Internet to make appointments can substantially reduce their waiting time. The key reason may be that using a pre-scheduling appointment system, doctors can regulate their service capacity and balance the demands between online and offline channels, which helps to avoid overload situations and reduce patients' meaningless wait.

Health- and cost-related risk factors influence patients' channel choice. Our results suggest that both health- and cost-related risk factors are the two critical major concerns of patients, which is consistent with prior studies (31, 32). These factors significantly improve the patients' propensity to make appointments *via* the Internet. The possible reason is that the Internet improves information transparency and disclosure, which helps patients reduce uncertainty. When patients have higher health- or cost-related risk factors, such as getting a serious disease, they tend to choose a channel with higher perceived accessibility and information quality (27).

**Figure 3:**
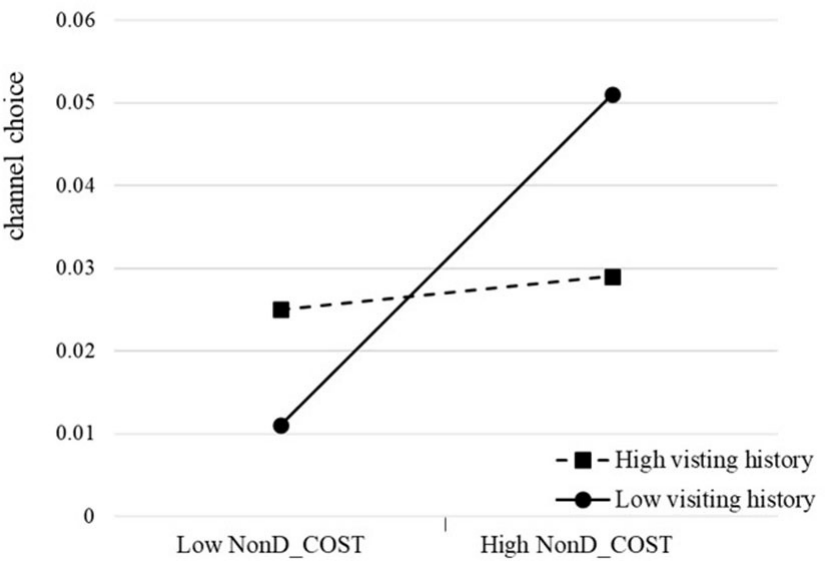
The moderating effect of visiting history on channel choice.

The moderating effects of patients' visiting history show heterogeneity. We further find that patients' visiting history only eliminates the positive relationship between the cost-related risk factor and channel choice (refer to Figure 3). No evidence has been found for his moderating effect on the relationship between health-related risk factors and channel choice. There are two possible explanations. First, compared with the cost-related risk factor, patients care about health-related risk factors more since medical services deal with life and wellness. High-quality medical services are valuable exchange resources and are greatly desired but scarce (48). As lacking relevant technical skills and professional medical knowledge, patients struggle to get high-quality medical resources *via* various means. The online channel helps patients get access to satisfied doctors. Second, the cost-related risk is easy to be measured compared with health-related risk. Thus, a channel with rich information is needed for patients to reduce their uncertainty. Therefore, even if patients have visiting history in the hospital, they still rely on the online channel to help reduce their perceived uncertainty.

### Implications

This study produces several insights, which have implications for medical process optimization, channel choice, and patient's behavior literature. More importantly, these insights contribute to the design of appointment systems in hospitals.

Our study contributes to knowledge in several ways. First, our work extends our knowledge of the impact of IT in health care from the perspective of waiting time. Patient's waiting time is closely related to their willingness to return for care and satisfaction ratings (19, 20) and affects the utilization of healthcare services (3). However, to date, there are few studies about the efficacy of online appointments on reducing patient's waiting time that are conducted using a big sample size and real operation data. Most existing studies depend on survey data (6). Our results show that by implementing an online appointment channel, patient's waiting time can be decreased significantly.

Second, our study provides evidence on channel choice in health care. To date, studies on channel choice mainly focus on other fields (49, 50), and the determinants of channel choice have not been fully understood in health care. By considering the special characteristics of medical services, we include both health-related and cost-related factors and find heterogeneity in the results. Examination of the two dimensions of factors allows us to understand channel choice more comprehensively.

Third, this study enriches patient's behavior literature under the “Internet plus healthcare” background. With the application of information technology in health care, researchers have extensively investigated patient's behavior in the online channel context (32, 51) and overlooked patient's behavior in the multi-channel context. Driven by policies on the “Internet plus healthcare,” a multi-channel strategy will be widely adopted. The findings of this study suggest that determinants of patient behavior can be divided into different dimensions and have different influences.

In practice, first, because of China's limited medical resources, long waiting time for consultation is common in the healthcare system and seriously influences the patient's experience. Understanding the impact factors of patient's waiting time helps administrators of hospitals take useful strategies to reduce waiting time and improve satisfaction. In particular, we provide practical insights into physicians and hospitals in the era of “Internet plus.” This study emphasizes the effect of information technology use on reducing patient's waiting time and suggests that hospitals can improve their efficiency by integrating the online channel. The contribution of this study to the healthcare system includes the following three aspects: first, it will improve the efficiency of healthcare services by optimizing the online and offline processes. Second, it reduces the waste of medical resources by reducing the occurrence of patients leaving the waiting area without being seen by a physician due to long waiting times. Third, by reducing offline waiting time, it reduces the aggravation of patients' conditions caused by unnecessary waiting time and reduces the additional burden on the healthcare system. The contribution of this study to patients includes the following two aspects: First, it will improve offline waiting time and reduce the cost of care for patients through multi-channel service. The second is to improve the patient's experience by reducing offline waiting time and anxiety in crowded environments.

Second, this study has revealed the determinants of online channel choice and found different effects. Based on our dataset, there only one-third of patients use the online channel to make appointments. There's still a lot of room for hospitals to develop the online channel. By identifying the determinants of patients' channel choice, hospitals can develop intervention strategies to further improve the usability of online channels. To facilitate the use of the online channel, the management of hospitals should set encouraging mechanisms to appeal to their patients to make appointments *via* Internet, such as putting more resources into developing online channels or establishing cooperative relationships with third-party platforms.

Third, based on our dataset, we find that there are 62.3% of patients want to see a doctor in the morning, which makes it very difficult to obtain appointments during this period. Hospitals can regulate it by introducing the distribution mechanism of appointment sources in the online channel to regulate demands evenly and encourage patients to make appointments during periods with a lower outpatient load.

### Limitations

Although this research has highlighted several notable findings and contributions, we acknowledge some limitations. First, we only obtained data from a hospital, and the results need to be cross-validated in other hospitals. Second, this study, as in most cross-sectional research, cannot infer causality and the dynamic effects. Future researchers should design longitudinal studies to replicate the research findings. Despite these potential limitations, our study demonstrates that the online appointment channel is an efficient means to reduce patient's waiting time. By identifying the determinants of patients' channel choice, hospitals can develop intervention strategies to further improve the usability of online channels.

### Conclusions

To the best of our knowledge, our study is among the first that tests the effects of appointment channels on patient's experience measured by patient's waiting time and investigates the determinants of channel choice in health care. By collecting real operation data from 1,241 doctors from 119 departments, involving 308,085 patients from a tertiary hospital in China, we find that first, the average patient's waiting time in the consulting room is 99.47 min, with 92.18 min for patients with online appointment channel and 114.55 min for ones with offline appointment channel. Second, our results confirm the effect of Internet use on reducing patient's waiting time. Patients consider both health-related risk factors and cost-related risk factors to make decisions on appointment channels. Third, the moderating effects of patients' visiting history show heterogeneity. Patients' visiting history only eliminates the positive relationship between the cost-related risk factor and channel choice, but no evidence has been found for his moderating effect on the relationship between health-related risk factors and channel choice. Because of China's limited medical resources, long waiting time for consultation is common in the healthcare system and seriously influence patient's experience. Our findings can help relevant people understand the effects of information technology on reducing patient's waiting time and these insights contribute to the designs of the appointment systems in hospitals.

## Data availability statement

The datasets used and/or analyzed during the current study are available from the corresponding author on reasonable request.

## Author contributions

HW is responsible for framework development, model design, and first draft writing. QY is responsible for data processing, model design, and review. All authors contributed to the article and approved the submitted version.

## Funding

This work was supported by the Natural Science Foundation of China (NSFC) (72001087).

## Conflict of interest

The authors declare that the research was conducted in the absence of any commercial or financial relationships that could be construed as a potential conflict of interest.

## Publisher's note

All claims expressed in this article are solely those of the authors and do not necessarily represent those of their affiliated organizations, or those of the publisher, the editors and the reviewers. Any product that may be evaluated in this article, or claim that may be made by its manufacturer, is not guaranteed or endorsed by the publisher.
